# *Plasmodium vivax* Protein PvTRAg23 Triggers Spleen Fibroblasts for Inflammatory Profile and Reduces Type I Collagen Secretion *via* NF-κBp65 Pathway

**DOI:** 10.3389/fimmu.2022.877122

**Published:** 2022-06-13

**Authors:** Hangye Zhang, Feihu Shen, Jiali Yu, Jieyun Ge, Yifan Sun, Haitian Fu, Yang Cheng

**Affiliations:** ^1^ Laboratory of Pathogen Infection and Immunity, Department of Public Health and Preventive Medicine, Wuxi School of Medicine, Jiangnan University, Wuxi, China; ^2^ Lianyungan Center for Disease Control and Prevention, Wuxi, China; ^3^ Department of Clinical Laboratory, Affiliated Hospital of Jiangnan University, Wuxi, China; ^4^ Department of Nuclear Medicine, Affiliated Hospital of Jiangnan University, Wuxi, China

**Keywords:** *Plasmodium vivax*, PvTRAg23, spleen fibroblasts, NF-κBp65 pathway, collagen

## Abstract

*Plasmodium vivax* is the most widespread human malaria parasite. The spleen is one of the most significant immune organs in the course of *Plasmodium* infection, and it contains splenic fibroblasts (SFs), which supports immunologic function by secreting type I collagen (collagen I). *Plasmodium* proteins have rarely been found to be involved in collagen alterations in the spleen during infection. Here, we selected the protein *P. vivax* tryptophan-rich antigen 23 (PvTRAg23), which is expressed by the spleen-dependent gene *Pv-fam-a* and is a member of the PvTRAgs family of export proteins, suggesting that it might have an effect on SFs. The protein specifically reduced the level of collagen I in human splenic fibroblasts (HSFs) and bound to cells with vimentin as receptors. However, such collagen changes were not mediated by binding to vimentin, but rather activating the NF-κBp65 pathway to produce inflammatory cytokines. Collagen impaired synthesis accompanied by extracellular matrix-related changes occurred in the spleen of mice infected with *P. yoelii* 17XNL. Overall, this study is the first one to report and verify the role of *Plasmodium* proteins on collagen in HSF *in vitro*. Results will contribute to further understanding of host spleen structural changes and immune responses after *Plasmodium* infection.

## Introduction

Malaria is a worldwide problem, and as many as 241 million *Plasmodium* infections occurred last year, which poses a serious threat to global public health (1). Between malaria etiological agents that infect humans, *Plasmodium vivax* is the most geographically widespread, and is recognized as a significant cause of morbidity and mortality in Central and South America and the Western Pacific (2). In human hosts, *P. vivax* infections result in fever, chills, anemia, splenomegaly, or severe malaria (3). Although clinical manifestations are similar between infected species, *P. vivax* leads to more obvious changes in the spleen (4). The spleen structure of the host changes significantly after infection, which can cause asymptomatic swelling or complications, such as rupture, ectopic spleen, hypersplenism, and hematoma formation (5). Therefore, the persistence and recurrence of *P. vivax* malaria seriously affect the life quality of patients and pose a real threat to their health.

The spleen serves several important roles during *Plasmodium* infection, ranging from induction of adaptive immune responses, production of erythrocytes, and selective filtration of aging and infected red blood cells (iRBCs) (6). Of note, splenic fibroblasts (SFs) are one of the main components supporting the structure and function of the spleen (7); which are widely distributed in connective tissues and help to deposit structural proteins such as collagen and elastin fibers into the extracellular matrix (ECM) (8, 9). Type I collagen (collagen I) is the major component of ECM secreted by fibroblasts and is crucial to create a microenvironment conducive to the development of immune responses in lymphoid organs (10). In the spleen, SFs secrete and remain associated with collagen I, they combine with argyophilic reticular fibers which strengthen the locules for filtration (11). Lymphocytes and myeloid cells are enclosed and orderly separated partly by collagen I and other adhesion proteins to ensure their normal operation (12, 13). During *Plasmodium* infection in rodents, iRBCs circulate in the blood, pass through the spleen to the filter beds, where they are shunted from small arteries to venous sinuses, and eventually cleared by immune cells in the filtration beds (14), while the specific circulation and clearance mechanism of iRBCs in the human spleen remains unclear. Normally, the spleen shows two types of circulation, open in rodents and closed in humans, respectively (15). However, upon infection with *P. yoelii* 17XNL, the open circulation in mouse spleen turns into a temporarily closed system due to the formation of a physical barrier made up of fibroblasts (16). In fact, after infected with *Plasmodium*, the SFs of both species will be abnormally activated and then evolve into barrier cells that interact with fibronectin and collagen I to form a blood–spleen barrier (16, 17). The barrier protects reticulocytes and erythroblasts in the red pulp from parasitization but allows iRBCs to adhere and realize immune escape (18, 19), which may sometimes lead to an imbalance of immune response and even cause severe diseases. Thus, understanding the changes of SFs and collagen I during *Plasmodium* infection is important.

Spleen-dependent genes of *Plasmodium* parasites depend on the spleen for transcription and translation (20). They exist in many *Plasmodium* species and may affect the spleen (20–23). In the study about *P. vivax* infections of spleen-intact and splenectomized *Aotus* monkeys, 67 spleen-dependent genes were identified by a global transcriptional approach (17). Among these, VIR proteins encoded by the *vir* multigene family exhibit different types of subcellular localization, and VIR14, belonging to the C subfamily, could adhere to SFs expressing ICAM-1 and mediate *Plasmodium* adhesion to it, whereas VIR members of subfamilies A and D, which do not have spleen dependence, could not achieve this adhesion (17, 19). This finding suggests that although the proteins are expressed by the same gene family, the presence or absence of spleen dependence may result in different functions.

Among all known spleen-dependent genes of *P. vivax*, five are from the *Pv-fam-a* gene family, and they express proteins known as *P. vivax* tryptophan-rich antigens (PvTRAgs) (17). PvTRAgs are a type of export proteins that are enriched in tryptophan residues (24) with high immunogenicity and conservation (25, 26), suggesting that they may play a role in the invasion and maturation of *P. vivax* parasites. Thus far, the function of PvTRAgs on the spleen has not been reported. Therefore, PvTRAg23 was identified from five spleen-dependent PvTRAgs to investigate whether it could act on spleen-related cells and affect the role of *Plasmodium* on the spleen.

In this study, we hypothesized that proteins encoded by *Pv-fam-a* genes whose expression is dependent on an intact spleen would act on SFs and affect the surrounding microenvironment, resulting in changes in the ECM structure such as collagen I, and PvTRAg23 was tested to confirm this conjecture. In addition, we used a mouse model of *Plasmodium* infection to identify the physiological significance of this change. Our study aids in further understanding of host spleen structural changes and immune responses after *Plasmodium* infection, and provides a new perspective for research of immunity in the spleen.

## Materials and Methods

### Cell Culture

Human splenic fibroblasts (HSFs) (ScienCell, California, USA) were maintained in Fibroblast Medium (ScienCell, USA) containing 2% fetal bovine serum (FBS), 1% fibroblast growth supplement (FGS), and 1% antibiotic solution (P/S) at 37°C in a humidified incubator with 5% CO_2_. For better growth of HSFs, the culture dishes were precoated with poly-L-lysine (ScienCell, USA) to charged their surfaces, which increases cell adhesion. The precoated culture wares were left in a 37°C incubator overnight, rinsed twice with sterile water, and then added with 10 mL of the complete medium to culture the cells. The cell passage was conducted when the culture reached 95% confluency.

### Production and Purification of Recombinant Proteins

Gene sequences of *pvtrag23* and *pvtrag26* were obtained from the PlasmoDB website (https://plasmodb.org/plasmo/app; accession no. *PVX_101515* and *PVX_112660*). Both sequences were derived from the Sal-1 strain of *P. vivax*; *pvtrag23* and *pvtrag26* were generated by DNA synthesis, and the gene fragments were then inserted into the pET30a expression vector (Tianlin Bio, Wuxi, China). This vector was added with thioredoxin and His-tag at the N- and C-terminal ends to enable easier purification and immunodetection using polyclonal antibodies against His-tag. *Escherichia coli* (*E. coli*) BL21 (DE3) pLysS cells (TransGen Biotech, Beijing, China) were used as the expression host. The proteins rPvTRAg23 and rPvTRAg26 were purified using a Ni-Sepharose column under nondenaturing conditions by a biotechnology company (YouLong Bio, Shanghai, China).

### Western Blot Analysis

HSFs (5 × 10^6^ cells) were cultured in a 37°C incubator until the density reached 80%, pretreated with or without rPvTRAg23 and rPvTRAg26 for 48 h. Then the cells were harvested and resuspended in protein lysis buffer to extract protein. All samples were boiled and separated on a 10% SDS-PAGE, transferred to a polyvinylidene difluoride (PVDF) membrane, and blocked in blocking buffer (1 × TBST, 5% milk powder) for 2 h at room temperature. The blot was washed and incubated with primary antibodies, such as His (1:1000, Abcam, Cambridge, USA), collagen I (1:1000, Abcam, Cambridge, USA), vimentin (1:1000, Abcam, Cambridge, USA), NF-κBp65 (1:1000, CST, Danvers, USA), phospho-NF-κBp65 (1:1000, CST, Danvers, USA), FAK (1:1000, CST, Danvers, USA), phospho-FAK (1:1000, CST, Danvers, USA), p38 MAPK (1:1000, CST, Danvers, USA), phospho-p38 MAPK (1:1000, CST, Danvers, USA), and GAPDH (1:1000, Abcam, Cambridge, USA) at 4°C overnight. The blots were washed and incubated for 1 h with secondary antibody conjugated to HRP (1:5000, Abcam, Cambridge, USA). Bands were visualized by ECL detection kit (NCM, Suzhou, China), and protein expression was quantified by Image J (National Institutes of Health, MD, USA).

### Animal Immunization With Recombinant Proteins

Five BALB/c female mice, six weeks old, were immunized with formulations containing 50 μg recombinant vimentin protein. Mice were injected abdominally with 200 μL of the formulation three times, with an interval of two weeks between each injection. The formulation consisted of protein and Freunds adjuvants (Sigma, Darmstadt, Germany). The proteins were emulsified in a constant volume of adjuvant and then injected into mice. Complete Freunds adjuvant was used for the first injection and incomplete Freunds adjuvant was used for the next two injections. Mice were euthanatized 14 days after the last immunization and serum samples were collected. Meanwhile, a total of 1.5 mg rPvTRAg23 protein was used to immunize rabbits (YouLong Bio, Shanghai, China) for the production of polyclonal antibodies.

### Indirect Immunofluorescence Assay (IFA)

The schizont stage-rich parasites of *P. vivax* were collected from malaria patients in Thailand. The slides smeared parasite infected blood were fixed with 4% paraformaldehyde for 5 min, dried, and blocked with PBS containing 5% non-fat milk at 37°C for 30 min (26). Then, slides were incubated with rabbit anti-PvTRAg23 antisera (1:50) and mouse anti-VIR antibody (1:100) for 2 h, washed three times with PBS and air-dried. Anti-VIR antibody was donated by Dr. Louis H. Miller (Laboratory of Malaria and Vector Research, National Institute of Allergy and Infectious Diseases, NIH). After primary antibody reactions, the samples were then treated with Alexa Fluor 488-conjugated donkey anti-rabbit IgG (1:500, Invitrogen, CA, USA), Alexa Fluor 568-conjugated goat anti-mouse IgG (1:500, Invitrogen CA, USA), and 4, 6-diamidino-2-phenylindole (DAPI) (1:1000, Invitrogen, CA, USA). The slides were then sealed with neutral resin, and visualized with confocal laser-scanning microscopy (Zeiss, Jena, Germany) under oil immersion. Images were edited using Adobe Photoshop CS5 (Adobe Systems, San Jose, CA, USA).

### RNA Extraction and Quantitative Real-Time PCR

Total RNA was extracted from isolated HSFs (5 × 10^6^ cells) or pulverized spleen according to the manufacturer’s instructions (ES Science, Shanghai, China). The resulting total RNA was used for reverse transcription (RT). Genomic DNA was removed by gDNA Remover Mix, and cDNA was produced by 5 × HiFiScript RT MasterMix according to the manufacturer’s protocol (CWBIO, Beijing, China). Each sample was assayed in triplicate. RT-PCR was performed with 2 × TB Green Premix Ex Taq II (Takara, Japan) in an LightCycler480 II apparatus (Roche, USA). The thermal cycling conditions were as follows: initial denaturation at 95°C for 30 s, followed by 40 cycles of PCR at 95°C for 5 s and 60°C for 30 s, then 95°C for 5 s and 60°C for 1 min, followed by 50°C for 30 s. GAPDH was used as an internal control, and the ratio of each target gene was determined. The primer sequences are presented in **Supplementary Table S1**.

### Enzyme-Linked Immunosorbent Assay

Soluble collagen I levels in the cell supernatant were quantified by ELISA kit following the manufacturer’ s instructions (Abclonal, Wuhan, China). HSFs (5 × 10^6^ cells) were cultured in the presence of with rPvTRAg23 and rPvTRAg26 in 50 µg/mL for 48 h. Cell-free culture supernatant was collected and analyzed for collagen I content. Each sample was assayed in triplicate at 450 nm, and the collagen I concentration in supernatant was expressed in pg/mL.

### Flow Cytometry

HSFs (5 × 10^6^ cells) were harvested in clean EP tubes and stained with rabbit anti-His antibody (1:300, Abcam, Cambridge, USA) or rabbit anti-Vimentin antibody (1:300, Abcam, Cambridge, USA) at 4°C for 30 min. The stained HSFs were washed twice with 1 × PBS and then treated with Alexa Fluor 647-conjugated goat anti-rabbit IgG (1:2000, Abcam, Cambridge, USA) at 4°C for 1 h with light protection. The labeled HSFs were subjected to flow cytometry analysis using FACScan flow cytometer (Becton Dickinson, Franklin Lakes, NJ, USA). For cells that needed to block the vimentin receptors, HSFs were incubated with mouse anti-vimentin antisera (1:10) for 2 h after collection, washed twice with 1 × PBS, and performed the above experiments. The percentage of His-positive or vimentin-positive cells was determined using the FlowJo software package.

### Animal Models

Five six-week-old wild BALB/c mice weighing 18–22 g were purchased from Cavens Laboratory Animal (Changzhou, China). Strains of *Plasmodium yoelii* 17XNL were obtained from the Jiangsu Institute of Parasitic Disease in Wuxi, China. Mice were randomly grouped and intraperitoneally injected with 1 × 10^5^ iRBCs in 200 μL of saline, prepared from frozen stock. Animal precautions and experimental processes were approved by Jiangnan University’s Animal Ethics Committee.

### Human Embryonic Kidney (HEK) 293T Culture and Transfection

293T cells were maintained in DMEM supplemented with 10% FBS (Gemini Bio-Products), 100 U/mL penicillin, and 100 μg/mL streptomycin in a humidified atmosphere containing 5% CO2 at 37°C. For immunoprecipitation, 293T cells were transfected with Lipofectamine (Yeasen, Shanghai, China) according to the protocol provided by the manufacturer and cultivated for 24 h. Briefly, cells were grown on 6 cm plates, transfected with 5 μg pEGFP-C1-Vimentin eukaryotic plasmid and 5 μg pEGFP-C1-Vector for 20 min, then returned to DMEM for 24 h. The transfection efficiency was observed under a Nikon Inverted Fluorescent Microscope (Nikon, Tokyo, Japan). After the cells were lysed by NP40 cell lysate (Beyotime, Shanghai, China), the expression of vimentin was verified by western blot.

### Pull-Down Assay

The His-tagged rPvTRAg23 protein was immobilized on Ni-NTA agarose (QIAGEN, Germany) to generate an affinity column. Briefly, 200 μg of purified His-tagged rPvTRAg23 was added to 500 μL of Ni-NTA-agarose, which was washed in cold 1 × PBS twice and 1 mL of equilibration buffer (50 mM NaH_2_PO_4_, 300 mM NaCl, 20 mM imidazole, adjust pH to 8.0 by adding NaOH) and incubated overnight at 4°C in a silent mixer. The Ni-NTA-agarose/rPvTRAg23 protein columns were washed in cold equilibration buffer and added with 200 μg of HSF membrane proteins, which were extracted using the Mem-PER Plus Membrane Protein Extractiont Kit (Thermo, Waltham, MA, USA) and incubated at 4°C overnight. After washing the beads with equilibration buffer three times, the proteins were eluted with 500 μL of the elution buffer (50 mM NaH_2_PO_4_, 300 mM NaCl, 250 mM imidazole, adjust pH to 8.0 by adding NaOH) and size fractioned by sodium dodecyl sulfate polyacrylamide gel electrophoresis.

To evaluate the interaction between PvTRAg23 and vimentin, His-pulldown assays were carried out. rPvTRAg23 protein (200 μg) were incubated with 500 μL anti-His Ni-NTA-agarose (QIAGEN, Germany) for 3 h at 4°C to immobilize on beads. The beads were collected using a magnetic separator and incubated separately with 500 μL lysate of 293T transfected pEGFP-C1-vimentin overnight at 4°C. After washing the beads with 20 volumes of equilibration buffer three times, the proteins were eluted with 500 μL of the elution buffer. Take 100 μL eluent and resuspended in 25 μL of 5 × SDS reducing loading buffer and boiled for 8 min. Supernatant was used for western blot analysis.

### Silver Staining and Mass Spectrometry

The rPvTRAg23-binding proteins were visualized by silver staining using the Fast Silver Stain Kit (Beyotime, Shanghai, China) according to the manufacturer’s instructions. The protein bands different from rPvTRAg23 in silver-stained gel were excised and cut into 1 mm pieces. Proteins were identified by mass spectrometry (Shanghai Applied Protein Technology, Shanghai, China).

For proteomics of the spleens, the infected and non-infected mouse spleens were extracted under sterile conditions. The spleens were ground thoroughly, lysed in lysis buffer, inactivated by autoclave sterilization, and processed for quantitative proteomic analysis (Jingjie PTM BioLab, Hangzhou, China).

### Sirius Red Staining

Collagen fiber specially type I in the spleen was assessed by Sirius Red tissue staining on paraffin-embedded sections. After the removal of paraffin with xylene, different concentrations of ethanol (100%, 90%, 80%, and 70%), and water, the tissue sections were incubated in Sirius Red reagent (Solarbio, Beijing, China) for 1 h, followed by washing with 0.5% acidic water, and sealed with neutral resin. All the stained sections were observed under ordinary optical microscope (OLYMPUS, Tokyo, Japan).

### Statistical Analysis

Data with normal distribution from more than two groups were compared using one‐way ANOVA, while differences between groups were analyzed by Student’s t-test. All statistical analyses were performed using GraphPad Prism 8 software (GraphPad, California, USA). *P-value*<0.05 was considered statistically significant for differences (*P<0.05, **P<0.01, ***P<0.001).

## Results

### The Expression and Localization of rPvTRAg23 and rPvTRAg26

**Figure 1:**
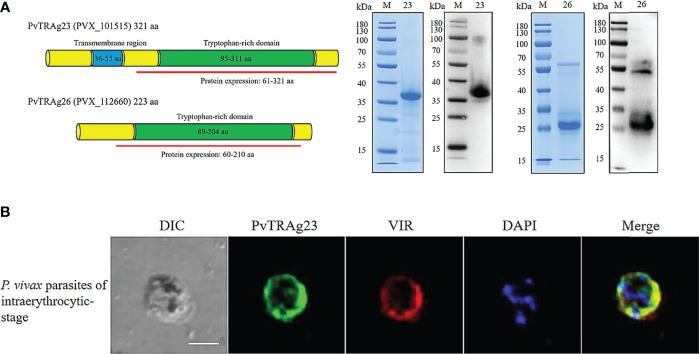
The Expression and localization of rPvTRAg23 and rPvTRAg26. **(A)** Gene sequences and domains of *pvtrag23* and *pvtrag26* were obtained from PlasmoDB. Fragments containing tryptophan-rich domains were selected for expression and linked to pET30a vector with His-tag (left). rPvTRAg23 and rPvTRAg26 proteins were purified from *E. coli* cells by Ni-Sepharose column under non-denaturing conditions. Purified rPvTRAg23 and rPvTRAg26 were separated by SDS-PAGE and stained with Coomassie blue, and the expressions of His-tag on recombinant proteins were confirmed by western blot analysis with anti-His antibody (right). M, Molecular size marker. 23, rPvTRAg23. 26, rPvTRAg26. **(B)** *P. vivax* parasites were probed with rabbit anti-PvTRAg-23 antisera (1:50) and mouse anti-VIR antibody (1:100), followed by Alexa Fluor 488-conjugated donkey anti-rabbit IgG (1:500, green) and Alexa Fluor 568-conjugated goat anti-mouse IgG (1:500, red). Parasite nuclei were stained with DAPI (blue). The bar represents 5 μm. DIC, bright-field images.

The full-length cDNA of the *PVX_101515* gene encodes the PvTRAg23 protein of 321 amino acids (aa) with molecular weight of 39.951 kDa and a predicted pI of 9.51, which contains a transmembrane region at residues 36–55 aa and a special tryptophan-rich domain residue at 95-311 aa. To elucidate the role of PvTRAg23, we expressed partial *PVX_101515* cDNA (encoding the tryptophan-rich domain) in *E. coli*. The recombinant PvTRAg23 (rPvTRAg23) of 36 kDa was purified to verify its role. Another recombinant protein rPvTRAg26 with high antigenicity and immunogenicity (27) was expressed for comparison **(** **Figure 1A** **)**. Studies on PvTRAgs have shown that many members of PvTRAgs are localized on the erythrocyte membrane. They could be exported through parasitophorous vacuolar (PV) or other ways to interact with host cells (26–29). To determine the location of rPvTRAg23, co-localization was carried out using anti-VIR antibody. VIR has previously been shown to be an export protein that could be exported outside the membrane of iRBCs and interact with host cells (19). Results of IFA assay on infected erythrocytes showed that rPvTRAg23 signal (green) partially merged with VIR (red) during the early or trophozoite stage of the parasite **(** **Figure 1B** **)**, indicating that rPvTRAg23 could be exported to the surface of iRBC and might interact with host cells.

### Collagen I Level in HSF Is Down-Regulated During rPvTRAg23 Stimulation

**Figure 2:**
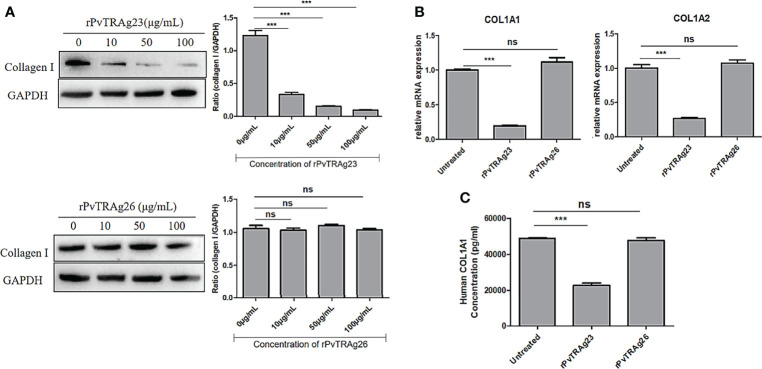
The levels of collagen I secreted by HSFs after stimulated by recombinant proteins. **(A)** 5 × 10^6^ HSFs were cultured with rPvTRAg23 and rPvTRAg26 at the corresponding concentration. After 48 h, the cells were lysed and the collagen I expression in HSFs was measured by western blot (left), the gray values were compared between the two groups using GAPDH as internal reference (right). **(B)** 5 × 10^6^ HSFs were treated with 50 μg/mL rPvTRAg23 or PvTRAg26 for 48 (h)Untreated cells were set for control, and collagen mRNA levels of cells were detected by qPCR. Three individual experiments have been performed. **(C)** Supernatants of HSFs culture were collected to detect collagen I content after 5 × 10^6^ HSFs were stimulated by 50 μg/mL rPvTRAg23 or rPvTRAg26 for 48 h. Each sample was assayed in triplicate at 450 nm and the collagen I concentrations in supernatant were expressed in pg/mL. Statistical analyses were carried out by one-way ANOVA with Dunnett’s multiple comparisons test. (ns, *P* > =0.05; ***P < 0.005).

Spleen section from a *P. vivax*-infected patient showed a large number of malaria parasites and hemozoin (30). Meanwhile, after *Plasmodium* infection, destruction of the segregated lymphoid areas of the white pulp and remodeling of the red pulp were found in the host spleens (6), suggesting that the clearance function of the spleen might be affected during this period. Collagen I secreted by SF is an important component in maintaining spleen struction and functions (10, 11), while VIR protein, expressed by the spleen-dependent gene of *P. vivax*, has been proved to interact with HSFs to achieve immune evasion (17). Thus, as PvTRAg23 was also expressed by spleen-dependent gene, it might play a role in this process. To investigate the function of PvTRAg23 on HSFs, the cells were cultured with different concentrations of recombinant protein for 48 h, and the levels of collagen I in cells were detected. With the increase of rPvTRAg23 concentration, the protein expression of collagen I in HSFs decreased, while control rPvTRAg26-stimulated cells had no significant effect on it **(** **Figure 2A** **)**, showing that rPvTRAg23 affected the collagen I content in HSFs specifically and in a concentration-dependent manner. Similar decreases in the mRNA levels of collagen I alpha 1 chain (COL1A1) and collagen I alpha 2 chain (COL1A2) were observed in response to protein stimulation **(** **Figure 2B** **)**. Given that collagen I is the main component of ECM, the level of collagen I in the supernatant of HSF culture medium was measured after 48 h of protein stimulation to verify whether there is a change in collagen outside the cells. The ELISA results showed that the collagen I level in the supernatant of rPvTRAg23-stimulated HSFs culture medium decreased significantly compared with that in untreated group **(** **Figure 2C** **)**. These data infered that rPvTRAg23 reduced the content of collagen I production by HSFs and thus affected its extracellular secretion.

### The Partial Blockage of the Binding Between rPvTRAg23 and HSF Vimentin Does Not Avoid Collagen I Reduction

**Figure 3:**
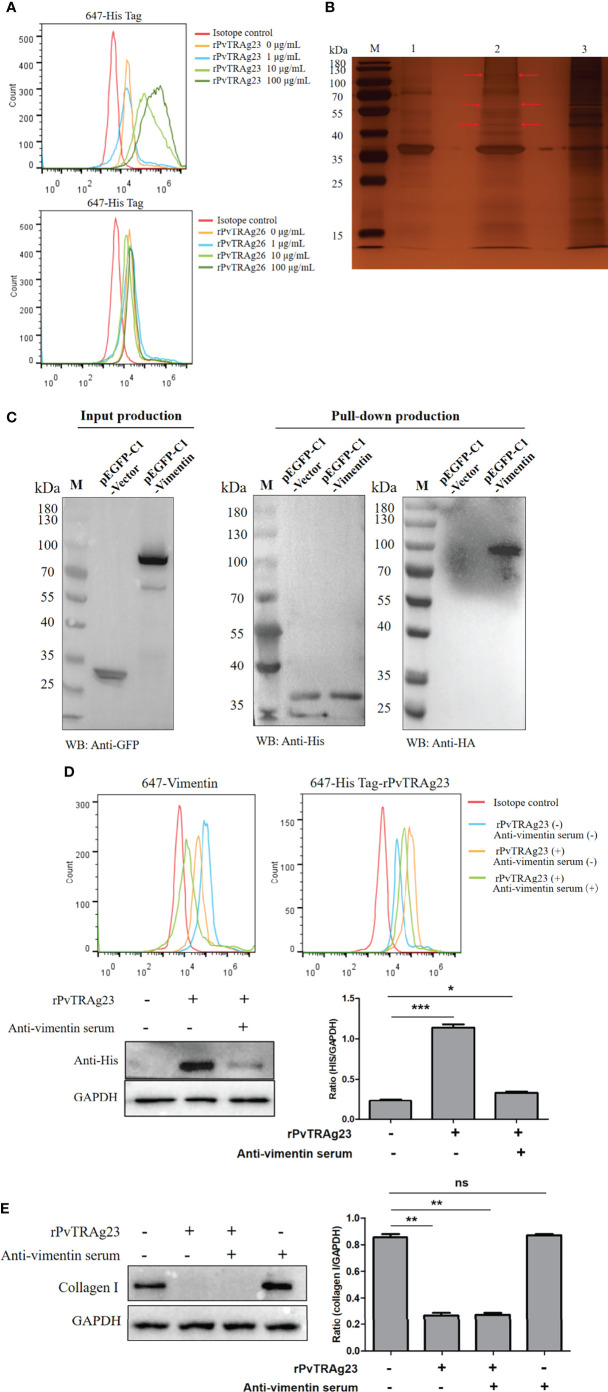
rPvTRAg23 binds to HSF vimentin. **(A)** 5 × 10^6^ HSFs were collected and the corresponding concentrations of rPvTRAg23 and rPvTRAg26 were added respectively. After mixed incubation for 3 h, the cells were stained with rabbit anti-His antibody, followd by Alexa Fluor 647-conjugated goat anti-rabbit IgG. Then, flow cytometry was used to analyze the fluorescence markers in each group. **(B)** 200 ng HSF membrane proteins were incubated with recombinant His-tagged PvTRAg23 on Ni-NTA Resin overnight, washed, eluted and separated on silver-stained SDS-PAGE gel. Red arrows are denoting unique bands. M, Molecular size marker; 1, rPvTRAg23 protein; 2, Eluent of HSF membrane proteins co-incubated with rPvTRAg23; 3, HSF membrane proteins. **(C)** After transfecting pEGFP-C1-vimentin and pEGFP-C1-vector eukaryotic plasmid with 293T cells for 24 h, the cells (3 × 10^6^) were lysed and the lysates were detected by western blot. The proteins expressed in cells (GFP-Tag) were verified by anti-GFP antibody (left). The 500 μL lysate and rPvTRAg23 were incubated on Ni-NTA Resin overnight, eluted and the elution of rPvTRAg23 protein was verified with anti-His antibody, while the binding of vimentin (HA-Tag) was verified with anti-HA antibody (right). **(D)** 5 × 10^6^ HSFs were pretreated with mouse anti-vimentin antisera (1:10). The treated and untreated groups were mixed with 50 μg/mL rPvTRAg23 respectively for 3 h, stained with rabbit anti-vimentin (1:200, up, left) and rabbit anti-His (1:200, up, right) antibodies and then incubated with Alexa Fluor 647-conjugated goat anti-rabbit IgG with light protection. The fluorescence intensity of each group was analyzed and compared by flow cytometry. Western blot verified the binding strength of vimentin receptor occluders (down, left), GAPDH was used as an internal parameter for gray value comparison (down, right). **(E)** Both mouse vimentin serum pretreated and untreated 5 × 10^6^ HSFs were stimulated with 50 μg/mL rPvTRAg23 for 48 h, the expression of collagen I in each group was detected compared with the untreated group, and GAPDH was used to participate in the gray value (right). Plus (+) indicates being processed; minus (-) indicates not being processed. Statistical analyses were carried out by one-way ANOVA with Dunnett’s multiple comparisons test. (ns*P* > =0.05; *P < 0.05; **P < 0.01; ***P < 0.001).

Many effects of *Plasmodium* on host cells are mediated by binding (31–33). As PvTRAg23 was an export protein, we analyzed its interaction with HSF. Flow cytometry analysis showed a high expression rate of His on HSFs in a dose dependent way of rPvTRAg23 simulation; meanwhile, no difference was found in the rPvTRAg26-stimulated HSFs group **(** **Figure 3A** **)**. This finding implied that rPvTRAg23 could specifically bind to HSFs. Thus, we expect to identify receptors for binding and explore whether it relates to collagen I reduction. The comparison of rPvTRAg23, HSF membrane protein, and the eluate after co-incubation showed differential bands when visualized by silver stain **(** **Figure 3B** **)**. The strips were cut off and analyzed by mass spectrum. Among the identified candidate proteins, vimentin yielded the highest score **(** **Supplementary Table S2** **)**, suggesting that it was likely to be the binding receptor of rPvTRAg23 and HSFs. Hence, 293T cells were transfected with pEGFP-C1-vimentin eukaryotic plasmid and the His-pulldown experiment was performed after verifying the expression of vimentin. As shown in **Figure 3C**, obvious interactions could be observed between rPvTRAg23 and vimentin. So we blocked the vimentin receptors on HSFs by anti-vimentin antisera prior to protein stimulation and performed the same test again. The flow cytometry results showed that the His expression rate decreased almost as much as that of the unstimulated group, similar to the western blot data **(** **Figure 3D** **)**. Hence, vimentin was indeed a binding receptor between rPvTRAg23 and HSFs. Furthermore, HSFs were stimulated with rPvTRAg23 after blocking vimentin receptors, and collagen I level was detected. The content of collagen I in the cells decreased regardless of whether the blocking of the vimentin receptors **(** **Figure 3E** **)**. In contrast to expectations, the binding of rPvTRAg23 to HSFs vimentin did not affect the reduction of collagen I in the cells. As such, we need to identify its mediated pathway from other perspectives.

### NF-κBp65 Pathway Mediates Increases in the Expression of IL-1β, IL-6, and TNF-α to Decrease Collagen I in HSF

**Figure 4:**
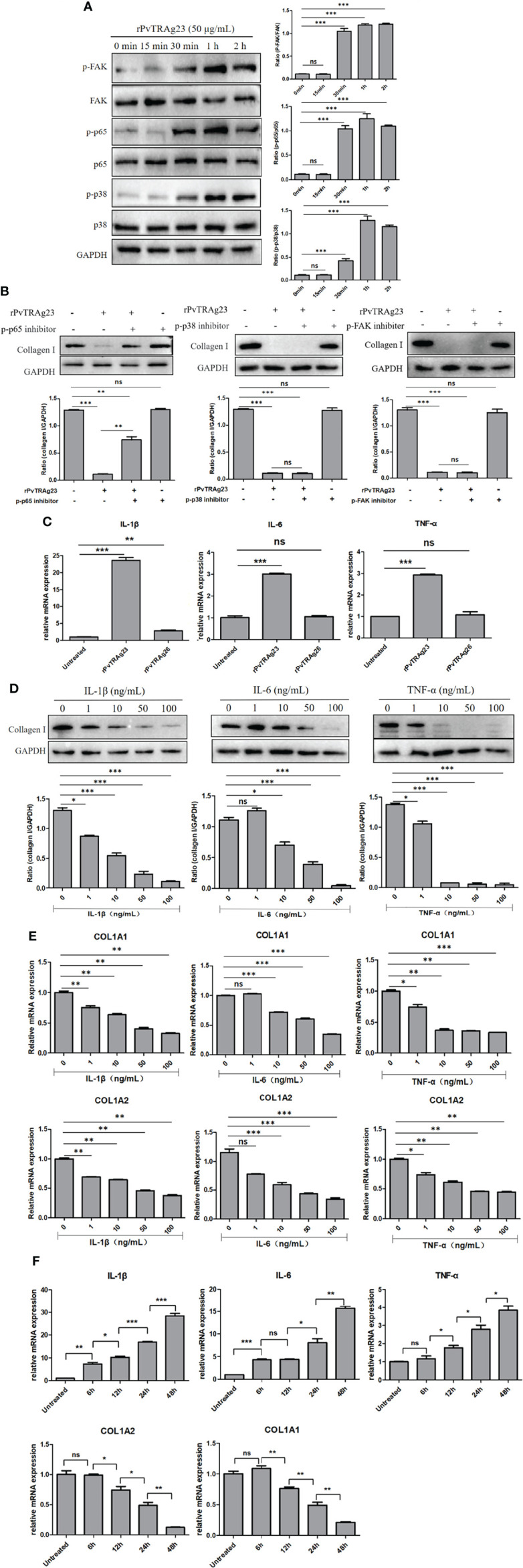
NF-κBp65 mediates increased expression of IL-1β, IL-6, and TNF-α to decrease collagen I in HSF. **(A)** 5 × 10^6^ HSFs were stimulated with 50 μg/mL rPvTRAg23 for corresponding time, the treated cells were collected and the corresponding signaling pathway antibodies were incubated respectively. The pathway activation status of each group was detected by western blot (left) and quantified by GAPDH as internal reference (right). **(B)** 5 × 10^6^ HSFs were pretreated with corresponding signaling pathway inhibitors for 1 h, and 50 μg/mL rPvTRAg23 was used to stimulate treated and untreated cells. After 48 h, the expression of collagen I in each group was detected by western blot (up), and GAPDH was used as the internal participating group for comparison (down). Plus (+) indicates added; minus (-) indicates not added. **(C)** 5 × 10^6^ HSFs were treated with 50 μg/mL rPvTRAg23 or rPvTRAg26 respectively, and RNA was extracted from the treated and untreated groups 48 h later. The expressions of related cytokines were detected by qPCR and compared with the control group. Three individual experiments have been performed. **(D)** The corresponding concentrations of commercial cytokines were used to incubate 5 × 10^6^ HSFs respectively for 48 h, lysed the cells and detected the expression of collagen I in each group by western blot (up). GAPDH was used as an internal parameter for gray value comparison (down). **(E)** 5 × 10^6^ HSFs were stimulated with the corresponding concentrations of IL-1β, IL-6 or TNF-α respectively for 48 h, RNA was extracted from each group. The mRNA expressions of collagen I were detected by qPCR and compared with that in the untreated group. Three individual experiments have been performed. **(F)** 5 × 10^6^ HSFs were stimulated with 50 μg/mL rPvTRAg23 for different times and the mRNA expression of each group was detected by qPCR. Three individual experiments have been performed. Statistical analyses were carried out by one-way ANOVA with Dunnett’s multiple comparisons test. (ns*P*>=0.05; *P < 0.05; **P < 0.01; ***P < 0.001).

Previous studies showed that NF-κBp65, FAK, and P38 MAPK signaling pathways can regulate collagen changes in cells and thus cause different reactions in the body (34–36). Thus, we explored whether rPvTRAg23 decreases the collagen I expression in HSFs through these signaling pathways. Here, HSFs were stimulated with rPvTRAg23, and each signal path state in different time periods was detected. The western blot results showed that the signal intensities of the three pathways increased with prolonged stimulus time **(** **Figure 4A** **)**. This finding suggested that rPvTRAg23 activated all the detected signaling pathways of HSFs. To determine which pathway mediates the reduction of collagen I in HSFs, we treated the cells with corresponding inhibitors, namely, Bay 11-7082, SB203580, and TAE226. After 1 h of pretreatment, the HSFs were stimulated with rPvTRAg23 and continued to culture for 48 h. The decline in the collagen I level was significantly improved after the inhibition of the NF-κBp65 pathway compared with the control group, while the level continued to decrease even after the inhibition of other pathways **(** **Figure 4B** **)**. Hence, the NF-κBp65, FAK, and P38 MAPK pathways of HSFs were activated in response to rPvTRAg23 stimulation, but only the NF-κBp65 pathway mediated intracellular collagen reduction.

The activation of the NF-κBp65 pathway is often accompanied by the increase in the levels of inflammatory cytokines, such as interleukin-1β (IL-1β), interleukin-6 (IL-6), and tumor necrosis factor-α (TNF-α) (37, 38). In this regard, we hypothesized that these cytokines might directly regulate collagen I expression in HSFs. The levels of relevant cytokines in HSFs after protein stimulation were measured. The rPvTRAg23-stimulated group showed significant increases in the levels of IL-1β, IL-6, and TNF-α compared with the other groups **(** **Figure 4C** **)**. To verify the role of cytokines on HSFs, we added different concentrations of commercial cytokines IL-1β, IL-6, and TNF-α to cells and cultured them for 48 h. Collagen I level was subsequently measured. With increasing cytokine concentration, the collagen I protein expression was reduced **(** **Figure 4D** **)**, similar to the mRNA level **(** **Figure 4E** **)**. Hence, these cytokines inhibited collagen I expression in HSFs in a concentration-dependent manner. To further verify the relationship between protein regulation of collagen I and inflammatory cytokines, HSFs were stimulated by rPvTRAg23 with time gradient, and the changes of cytokines and collagen I were detected at the same time. The results showed that with the increase of stimulation time, the level of inflammatory cytokines increased while collagen I decreased, and the two were negatively correlated **(** **Figure 4F** **)**, showed that the regulation of collagen I in HSFs by rPvTRAg23 had a certain relationship with inflammatory response. Overall, rPvTRAg23 stimulation activated the NF-κBp65 pathway in HSFs and thus increased the expression of inflammatory cytokines IL-1β, IL-6, and TNF-α, thereby inhibiting collagen I production.

### Collagen I and Collagen VI in the Spleen of *P. yoelii* 17XNL-Infected Mice Are Decreased

**Figure 5:**
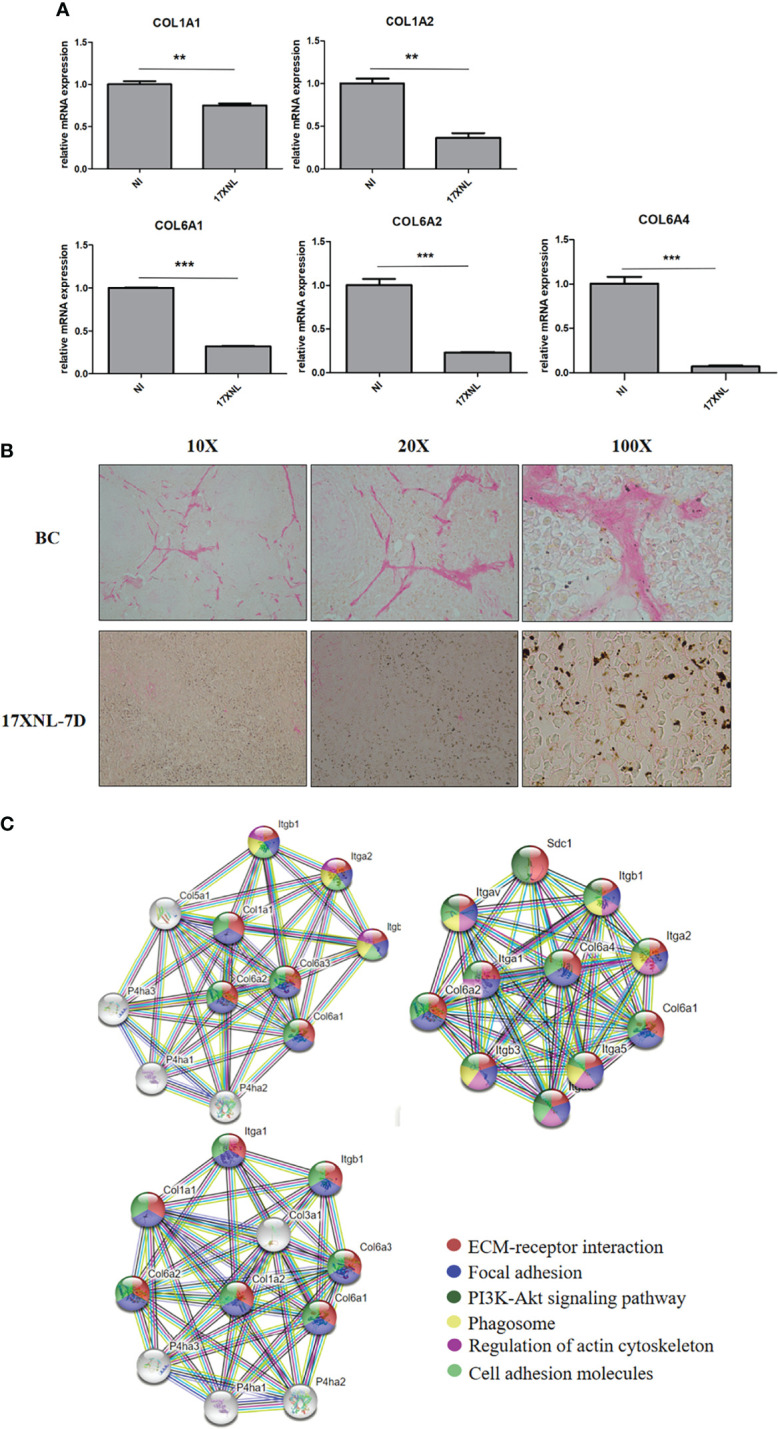
Collagen levels in spleens of *P. yoelii* 17XNL-infected mice. **(A)** 200 μL saline containing 1 × 10^5^ iRBCs was intraperitoneally injected into five BALB/C mice, and the same amount of PBS was injected into the other five mice. After 9 days, the mRNA expression levels of collagen in spleens of the two groups were determined and compared. NI, non-infected mice spleen. 17XNL, *P. yoelli* 17XNL -infected mice spleen. **(B)** Spleen paraffin sections of untreated mice and the mice infected with *P. yoelii* 17XNL were prepared. After 1 h of sirius red staining, dehydrated, transparent and sealed, the staining of collagen I (red) in each group was observed under ordinary optical microscope. NI, non-infected mice spleen. 17XNL-7D, spleen of mice infected with *P. yoelii* 17XNL for 7 days. **(C)** Col6a1, Col6a2 and Col6a4 interaction networks were constructed in STRING. The edges between the nodes represent different sources of active interaction. KEGG pathway analysis was integrated into STRING, and the different colors of each node represented different pathways related to the protein. False discovery rates of all pathways were lower than 0.01, indicating that KEGG pathway analysis was significantly enriched. Statistical analyses were carried out by Student’s t-test. (**P < 0.01; ***P < 0.001).

Previous studies have shown that parasite deposition and circulatory changes in the spleen of mice infected with *P. yoelii* 17XNL are similar to those of patients with *P. vivax* (30, 39). Therefore, to investigate whether the collagen reduction occurred in the course of *Plasmodium* infection *in vivo*, we explored physiological ECM changes in mice models of infection with the *P. yoelii* 17XNL strain. Then, the spleens were analyzed by LC-MS/MS. The proteomic analysis showed that compared with non-infected mice, collagen VI, but not collagen I, was markedly reduced in the spleens of *P. yoelii* 17XNL-infected mice **(** **Supplementary Table S3** **)**. Collagen VI is a protein widely distributed in a variety of tissues and mediates the formation of fibronectin in the ECM of cultured fibroblasts (40). GO annotation analysis was performed to further understand the role of collagen VI. The result revealed that it was involved in cell and ECM composition, immune response, and cell adhesion **(** **Supplementary Table S4** **)**, and had similar functions to that of collagen I. To explore whether collagen I changes, we detected the collagen mRNA levels in the spleens of mice. Compared with the non-infected group, COL1A1 and COL1A2 in the spleen of *P. yoelii* 17XNL-infected mice had a downward trend, while the decline in collagen VI alpha 1 chain (COL6A1), collagen VI alpha 2 chain (COL6A2), and collagen VI alpha 4 chain (COL6A4) were more obvious **(** **Figure 5A** **)**. The spleen sections of mice were stained with sirius red to visualize collagen I. The expression of collagen fiber in the white pulp of infected mice spleen was greatly reduced under ordinary optical microscope, accompanied by a large amount of malaria pigment deposition **(** **Figure 5B** **)**. Overall, the collagen I level in the spleen of *Plasmodium*-infected mice was indeed reduced. Basing on the possible protein interaction between collagen I and collagen VI, we conducted STRING analysis (https://string-db.org/). The interaction maps of COL6A1, COL6A2, and COL6A4 showed that they interacted with COL1A1 and COL1A2 and co-regulated the components of ECM and the adhesion of cells **(** **Figure 5C** **)**. Meanwhile, they were highly interactive with proline hydroxylase, thereby maintaining collagen structure stability and adhesion receptors, such as integrins.

## Discussion

During *Plasmodium* infection, the spleen is the main site for immune response and elimination of iRBCs. The body produces significant splenic reactions, including splenomegaly (41) and unexplained spleen rupture (4), indicating the spleen is active during this period. Collagen I produced by SFs is the main component of the ECM in the spleen, and it interacts with SFs to form reticular fibers and supports the blood filtration function of the spleen. Here, to understand the role of PvTRAg23, a protein encoded by the spleen-dependent gene *Pv-fam-a*, on SFs, we expressed a recombinant protein rPvTRAg23 to interact with HSF. The protein activated the NF-κBp65 pathway and increased the level of related inflammatory factors, thereby reducing collagen I expression of HSFs and then might affect the ECM structure of the host spleen.

Of all proteins expressed by *P. vivax* spleen-dependent genes, VIR have been proved to have different functions and can interact with SFs (17). As export proteins with splenic-dependent, PvTRAgs might also play an important role. Therefore, we selected PvTRAg23 and constructed the rPvTRAg23 protein for experimental purposes. A study on the spleen of *P. yoelii* 17XL-infected mice found that a large number of proteins were synthesized and secreted following the activation of reticular cells, and collagen production may be suspended (11). Thus, we detected changes in the levels of collagen I in cells and the culture medium supernatant after rPvTRAg23 stimulation of HSFs. The results showed that they decreased strongly, while rPvTRAg26, without spleen dependence, did not achieve this reduction **(** **Figures 2A–C** **)**. This finding indicated that PvTRAg23-mediated collagen I reduction in HSF was specific. Collagen I is secreted by fibroblasts, supports the filtration and clearance function of the spleen, and keeps the spleen structurally intact. In tissues, the decrease in collagen I is often accompanied by cell migration (42), inflammatory response, and neutrophil increase (43). Based on the reduction of collagen I in HSF, PvTRAg23 is likely to cause inflammatory responses in the spleen and degradation of the ECM, thereby affecting the immune response capacity of the spleen. Given that only one spleen-dependent PvTRAg protein was selected for the experiment, the function of four other proteins remains to be clarified.

To verify how PvTRAg23 acts on HSF, we performed an interaction experiment and found it bound specifically to HSFs, further mass spectrometry revealed that vimentin was its primary binding receptor **(** **Figure 3B** and **Supplementary Table S2** **)**. Vimentin is a member of the type III intermediate filament proteins and is mainly expressed in cells of mesenchymal origin (44). Vimentin is involved in maintaining cell shape and integrity, supporting organelle anchoring, cytoskeletal interactions, and signal transduction (45). In the process of infection with human immunodeficiency virus, severe acute respiratory syndrome coronavirus, and other viruses, vimentin often acts as a co-receptor, enabling them to achieve effective invasion of cells (46–48). Studies of human lung fibroblasts also found that vimentin specifically interacts with COL1A1 and COL1A2, thereby affecting collagen I protein levels (49). Therefore, we initially hypothesized that PvTRAg23 acts on HSF through vimentin, resulting in collagen I degradation, but contrary to expectations this was not the case. After blocking the vimentin receptors on HSFs, the cells were stimulated with rPvTRAg23 again and collagen I was greatly reduced, suggesting that vimentin was not the direct agent of the protein’s action on HSF **(** **Figure 3D** **)**. Considering that vimentin also plays an important role in cell adhesion and spreading (50, 51), we speculated that the binding of PvTRAg23 to HSF might be related to the adhesion of *P. vivax* to SFs, but it will not be discussed further here.

**Figure 6:**
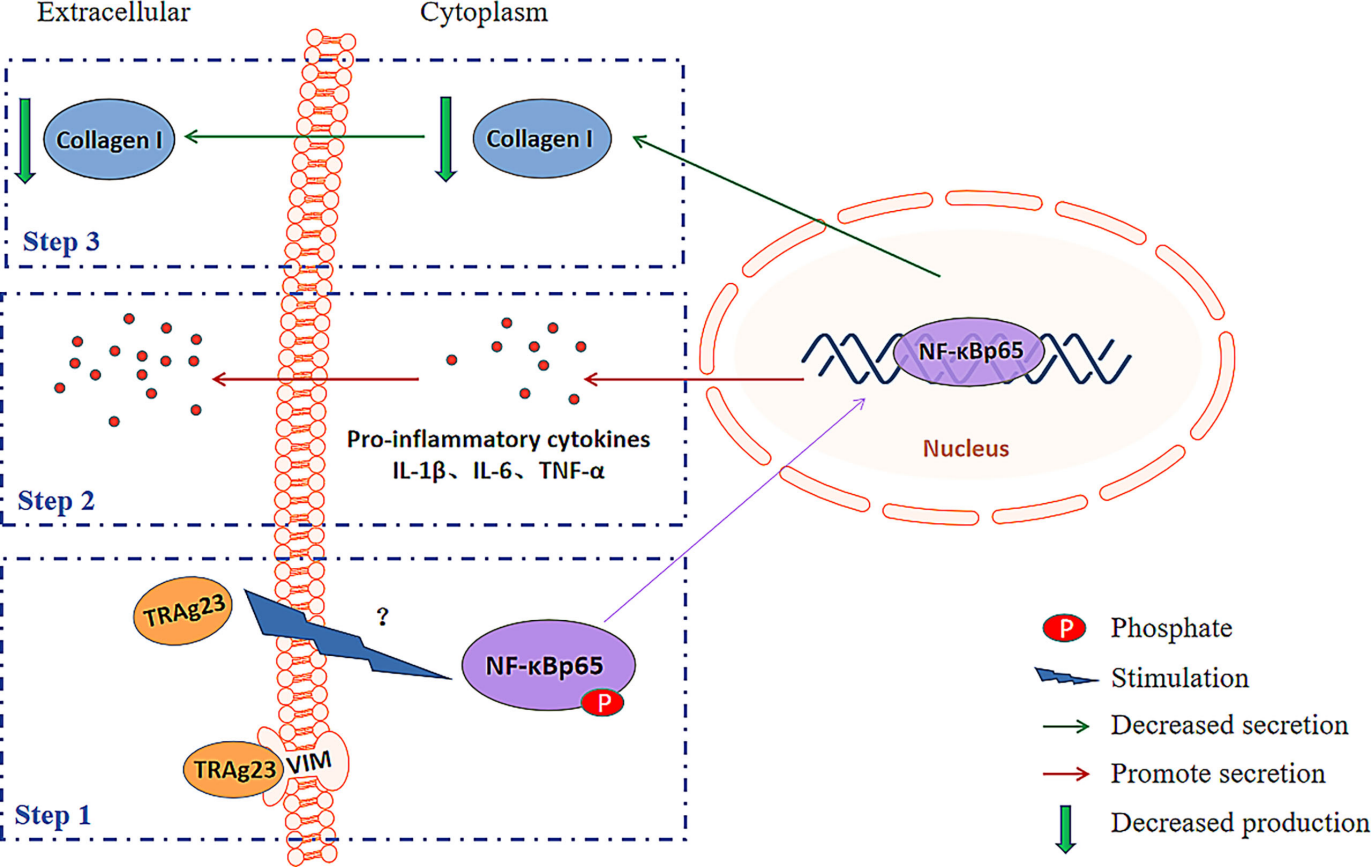
Schematic diagram explaining the possible mechanism for the collagen-remodeling induced by PvTRAg23-stimulated HSF.

Previous studies showed that changes in collagen I in cells and ECM are often accompanied by the activation of NF-κBp65 (35), FAK (52), and P38 MAPK (53) signaling pathways. In this regard, we detected the activation of pathways after rPvTRAg23 stimulation and collagen I changes after the addition of pathway inhibitors. We found that all three pathways were activated, but only NF-κBp65 mediated collagen I reduction in HSFs **(** **Figures 4A, B** **)**. Based on the important role in the expression of proinflammatory genes, the NF-κB pathway has long been considered a typical proinflammatory signaling pathway, and the heterodimer of p65 (RelA) is one of the most abundant forms (54). Inducible NF-κBp65 phosphorylation can occur in the cytoplasm and nucleus in response to a variety of stimulation; transcription of many inflammatory cytokines is upregulated during this process (55). TNF-α, IL-6, and IL-1β are proinflammatory cytokines in the NF-κBp65 pathway, and they perpetuate the inflammatory cascades (56). In the present research, the stimulation of rPvTRAg23 significantly increased the levels of inflammatory cytokines TNF-α, IL-6, and IL-1β in HSF, and this changes in cytokine level was negatively correlated with collagen I of HSF **(** **Figures 4C, F** **)**, which may be related to the activation of the NF-κBp65 pathway. In diabetic rat cardiomyocytes, the levels of TNF-α, IL-6, and IL-1β were negatively correlated with the mRNA expression of collagen I (57). Hence, these inflammatory cytokines may regulate collagen expression. Our subsequent experiments with commercial cytokines demonstrated this finding. All these data supports a model **(** **Figure 6** **)** in which PvTRAg23 activates the NF-κBp65 pathway in HSF, induces the release of pro-inflammatory cytokines TNF-α, IL-6, and IL-1β, and then affects the mRNA coding and protein production of collagen I. Ultimately, the collagen I level secreted outside of the cell decreased, thereby affecting the structure of ECM. In the current results, the activation mechanism of NF-κBp65 signaling pathway by PvTRAg23 was unknown. Due to the fact that some molecules could also regulate the activation of NF-κBp65 pathway through the nonclassical nuclear import of NF-κB essential modulator (NEMO) (58–60), we hypothesized that PvTRAg23 might act as a signal to activate the IKK complex in cells, leading to the ubiquitination of NEMO and indirectly activating the NF-κBp65 pathway. Although how the rPvTRAg23 activates the NF-κBp65 pathway remains to be determined, our research confirmed that the occurrence of inflammation in tissues is likely to impair the production of ECM, such as collagen I.

Given that the accumulation of parasites in the red pulp and the changes in the circulatory system in the spleen of *P. yoelii* 17XNL-infected mice are similar to those of *P. vivax* (30, 39), we used a mouse malaria model infected with *P. yoelii* 17XNL. In addition to collagen I, the level of collagen VI in the spleens of mice was evidently reduced. Collagen VI microfibrils are present in the ECM of almost all tissues, and they are closely associated with the basement membrane in the kidney, nerves, skin, and blood vessels (61). In mouse and human fibroblasts, the absence of collagen VI secretion affects the arrangement of fibronectin in the ECM, which could in turn trigger intracellular events, leading to myopathic defects (40). In the present study, the decrease in collagen VI in the spleen of infected mice indicated that the muscle tissue was likely damaged **(** **Figure 5A** and **Supplementary Table S3** **)**, which also provided a new angle to explain the cause of spleen rupture in human after infection with *Plasmodium*. The analysis of the spleen sections showed significant degradation of collagen I in the white pulp, a key component supporting the structure of the spleen. This finding also explains why the T- and B-cell regions of the white pulp of the spleen are disorganized after *Plasmodium* infection (62–64). The protein–protein interaction analysis revealed that collagen VI was highly interactive with collagen I, and both were involved in mediating multiple ECM-related functional pathways **(** **Figure 5C** **)**. As such, in the process of *Plasmodium* infection, regulation of ECM components, such as collagens, and functional interactions between host factors and ECM proteins might affect host responses and pathological results. Overall, the findings in mice showed that collagen degradation occurred in the spleens of *Plasmodium*-infected hosts and might influence the structure of the ECM in the spleen, thereby affecting its clearance of iRBCs.

In conclusion, PvTRAg23, with spleen dependence, mediated a decrease in collagen I in HSF, while *Plasmodium*-infected hosts show a reduction in type I and VI collagen levels in their spleens. These findings indicated that the effect of PvTRAg23 on HSF has physiological significance. Given that the immunologic function of the spleen such as clearing and filtering is dependent on the collagen-supported network, this effect may result in the destruction of the spleen’s ability to filter blood, allowing the parasite to escape.

## Data Availability Statement

The original contributions presented in the study are included in the article/**Supplementary Material**. Further inquiries can be directed to the corresponding author.

## Ethics Statement

The animal study was reviewed and approved by JN. no. 20200530b0301031.

## Author Contributions

YC conceived and designed the study. HZ, FS, and JG performed the experiments and analyzed the data. HZ and JY wrote the manuscript. YS and HF assisted in data processing and analysis and reviewed the manuscript. All authors read and approved the final version of the submitted manuscript.

## Funding

This work was supported by the National Natural Science Foundation of China (81871681).

## Conflict of Interest

The authors declare that the research was conducted in the absence of any commercial or financial relationships that could be construed as a potential conflict of interest.

## Publisher’s Note

All claims expressed in this article are solely those of the authors and do not necessarily represent those of their affiliated organizations, or those of the publisher, the editors and the reviewers. Any product that may be evaluated in this article, or claim that may be made by its manufacturer, is not guaranteed or endorsed by the publisher.
