# Supplementary material for: Plasmodium vivax Protein PvTRAg23 Triggers Spleen Fibroblasts for Inflammatory Profile and Reduces Type I Collagen Secretion via NF-κBp65 Pathway
Source: Front Immunol. 2022 Jun 13;13:877122. doi: 10.3389/fimmu.2022.877122 (PMC9235351; doi:10.3389/fimmu.2022.877122)
Supplement: Supplementary file 1 [file Table_1.docx]

**Table S1 |** Sequences of the primers used for HSF and mice spleen.

| **Gene name** | **Primer forward (5’−3’)** | **Primer Reverse (3’−5’)** |
| --- | --- | --- |
| IL-1β | ATGATGGCTTATTACAGTGGCAA | GTCGGAGATTCGTAGCTGGA |
| IL-6 | CCTGAGAAAGGAGACATGTAACAA | GGCAAGTCTCCTCATTGAATCC |
| TNF-α | CCCAGGCAGTCAGATCATCTTCT | ATGAGGTACAGGCCCTCTGAT |
| COL1A1 | GAGGGCCAAGACGAAGACATC | CAGATCACGTCATCGCACAAC |
| COL1A2 | AAAACATCCCAGCCAAGAACTG | TCAAACTGGCTGCCAGCAT |
| COL1A1-M | GCTCCTCTTAGGGGCCACT | ATTGGGGACCCTTAGGCCAT |
| COL1A2-M | GGTGAGCCTGGTCAAACGG | ACTGTGTCCTTTCACGCCTTT |
| COL6A1 | CTGCTGCTACAAGCCTGCT | GCACGAAGAATAGATCCACAGGG |
| COL6A2 | CCAGTCAACGTGTATTTCGTGT | TAGGCTCTTAGTGAAGGAGGC |
| COL6A4 | CTTCTGACAGCCGTACTGCC | CGAGCACGAATAATGCGATGC |
| GAPDH | CCTGCCTCTACTGGCGCTGC | GCAGTGGGGACACGGAAGGC |
| GAPDH-M | AGGTCGGTGTGAACGGATTTG | GGGGTCGTTGATGGCAACA |
