# Supplementary material for: Plasmodium vivax Protein PvTRAg23 Triggers Spleen Fibroblasts for Inflammatory Profile and Reduces Type I Collagen Secretion via NF-κBp65 Pathway
Source: Front Immunol. 2022 Jun 13;13:877122. doi: 10.3389/fimmu.2022.877122 (PMC9235351; doi:10.3389/fimmu.2022.877122)
Supplement: Supplementary file 2 [file Table_2.docx]

**Table S2 |** The results of silver stain gel identified by mass spectrometry

| **Protein No.** | **Accession Number** | **Protein name** | **Gene name** | **Mol. Wt[kDa]** | **Peptide identified** | **Score** |
| --- | --- | --- | --- | --- | --- | --- |
| 1 | P08670 | Vimentin | VIM | 53.6 | 9 | 79 |
| 2 | G1DUW4 | Truncated MHC class I antigen | HLA-A | 6.5 | 1 | 66 |
| 3 | B1B1G1 | Myelin proteolipid protein | PLP1 | 11 | 1 | 48 |
| 4 | K7ERB2 | AP-2 complex subunit beta | AP2B1 | 17.2 | 1 | 48 |
| 5 | A0A2R8Y4Q3 | Dynamin-like protein | OPA1 | 46 | 3 | 46 |
| 6 | HOYIVO | Endoplasmin | HSP90B1 | 17 | 2 | 46 |
| 7 | E5RJ73 | 28S ribosomal protein S27 | MRPS27 | 10.8 | 1 | 39 |
| 8 | I3L3P7 | 40S ribosomal protein | RPS15A | 11.5 | 1 | 34 |
