# Supplementary material for: Plasmodium vivax Protein PvTRAg23 Triggers Spleen Fibroblasts for Inflammatory Profile and Reduces Type I Collagen Secretion via NF-κBp65 Pathway
Source: Front Immunol. 2022 Jun 13;13:877122. doi: 10.3389/fimmu.2022.877122 (PMC9235351; doi:10.3389/fimmu.2022.877122)
Supplement: Supplementary file 3 [file Table_3.docx]

**Table S3 |** Abundance of Collagen in *P. yoelli* 17XNL-infected mice..

| **Protein accession** | **Gene name** | **Fold change** | **P-value** | **Mol. Wt [kDa]** | **Sequence coverage [%]** | **MS/MS Counts** |
| --- | --- | --- | --- | --- | --- | --- |
| Q04857 | Col6a1 | 0.272 | 4.4254E-10 | 108.49 | 29.8 | 377 |
| Q02788 | Col6a2 | 0.306 | 7.5778E-10 | 110.33 | 27.4 | 258 |
| A2AX52 | Col6a4 | 0.171 | 1.9608E-09 | 250.8 | 11.2 | 85 |
