# Supplementary material for: Plasmodium vivax Protein PvTRAg23 Triggers Spleen Fibroblasts for Inflammatory Profile and Reduces Type I Collagen Secretion via NF-κBp65 Pathway
Source: Front Immunol. 2022 Jun 13;13:877122. doi: 10.3389/fimmu.2022.877122 (PMC9235351; doi:10.3389/fimmu.2022.877122)
Supplement: Supplementary file 4 [file Table_4.docx]

**Table S4** | Distribution of differentially expressed collagen in GO secondary classification.

| **GO Terms Level 1** | **GO Terms Level 2** | **Collagen type** | | |
| --- | --- | --- | --- | --- |
| Biological Process | cellular process | Col6a1 | Col6a2 | Col6a4 |
|  | single-organism process | Col6a1 | — | — |
|  | response to stimulus | Col6a1 | Col6a2 | — |
|  | multicellular organismal process | Col6a1 | — | — |
|  | cellular component organizatio | Col6a1 | Col6a2 | Col6a4 |
|  | developmental process | Col6a1 | — | — |
|  | biological adhesion | Col6a1 | Col6a2 | Col6a4 |
| Cellular Component | cell | Col6a1 | Col6a2 | — |
|  | organelle | Col6a1 | Col6a2 | — |
|  | membrane | Col6a1 | Col6a2 | — |
|  | extracellular region | Col6a1 | Col6a2 | Col6a4 |
|  | macromolecular complex | Col6a1 | Col6a2 | Col6a4 |
| Molecular Function | binding | Col6a1 | — | — |
